# Carbonic Anhydrase as Pollution Biomarker: An Ancient Enzyme with a New Use

**DOI:** 10.3390/ijerph9113965

**Published:** 2012-11-01

**Authors:** Maria Giulia Lionetto, Roberto Caricato, Maria Elena Giordano, Elisa Erroi, Trifone Schettino

**Affiliations:** Department of Biological and Environmental Sciences and Technologies (Di.S.Te.B.A.), University of Salento, Via prov.le Lecce-Monteroni, Lecce 73100, Italy; Email: roberto.caricato@unisalento.it (R.C.); elena.giordano@unisalento.it (M.E.G.); elisaerroi@libero.it (E.E.); trifone.schettino@unisalento.it (T.S.)

**Keywords:** carbonic anhydrase, biomarker, heavy metals, pesticides, biomonitoring

## Abstract

The measurement of cellular and sub-cellular responses to chemical contaminants (referred to as biomarkers) in living organisms represents a recent tool in environmental monitoring. The review focuses on carbonic anhydrase, a ubiquitous metalloenzyme which plays key roles in a wide variety of physiological processes involving CO_2_ and HCO_3_^−^. In the last decade a number of studies have demonstrated the sensitivity of this enzyme to pollutants such as heavy metals and organic chemicals in both humans and wildlife. The review analyses these studies and discusses the potentiality of this enzyme as novel biomarker in environmental monitoring and assessment.

## 1. Introduction

In recent years environmental chemical contamination has increased considerably as a consequence of anthropogenic activities. For example chemicals such as heavy metals, oil based products, pesticides, fertilizers, plastic materials [1,2,3,4] have become of global concern for their impact on acquatic and terrestrial environments. For this reason the need to develop new methodological approaches for the identification, assessment and management of the risks for biota caused by chemical pollutants discharged to the environment has grown widely. The requirement for an integrated chemical and biological approach in environmental monitoring has received increasing attention. There is a growing awareness that focusing only on chemical data of pollutant concentration in environmental matrices (water, sediments, and soil) is insufficient to reliably assess the potential risks of the complex mixture of contaminants on biota. In fact, numerous environmental factors influence the bioavailability of pollutants to organisms such as temperature fluctuations, rainfall, pH, salinity, sediment type. In addition, numerous chemicals can be present simultaneously in the environment and chemical interactions in a mixture can cause complex and substantial changes in the chemical properties of pollutants, including bioavailability and toxicity. In general the adverse effects of a mixture of chemicals may not correspond to those predicted from data on pure chemical compounds.

Therefore, the need to also detect the biological effects of chemical contaminants at low concentrations and in complex mixture has increased the study of the relationships between exposure to chemical contaminants and alterations in several molecular and cellular processes in the organisms in order to use the latter as markers (commonly referred to as biomarkers) of exposure and early response to chemical contaminants. Biomarkers are defined as “pollutant-induced variations in cellular or biochemical components occurring in organisms as a result of natural exposure to contaminants in their environment” [5]. In environmental biomonitoring biomarkers are classified as biomarkers of exposure and biomarkers of effect. Biomarkers of exposure are early reversible cellular change in the organism and offer an early signal for exposure to micropollutants. They can be specific for single classes of pollutants. Biomarkers of effect give an assessment of physiological effects on the organisms and are directly related to the risk of adverse health effects. Since the harmful effects of pollutants are typically manifested at lower levels of biological organization before disturbances are realized at the population, community or ecosystem levels, the use of biomarkers measured at the molecular or cellular level has been proposed as sensitive “early warning” tools for biological effect measurement in environmental quality assessment [6]. As reported by several authors, the evaluation of biomarkers in bioindicator organisms sampled in one or more areas suspected of chemical contamination and their comparison with organisms sampled in a control area can allow the evaluation of the potential risk of toxicological exposure of the studied community [7,8]. The biomarker approach has been increasingly used in the last 20 years for the ecotoxicological assessment of marine, terrestrial and fresh water ecosystems. 

Because of the increasing attention towards the development of environmental “diagnostic” tools for early warning detection of pollution, in recent years the study of molecular and cellular effects of pollutants has given important advancement in the developing of biologically-based methodologies useful for environmental biomonitoring and risk assessment. 

In this field biochemical alterations such as enzymatic inhibition are attractive as indicators of environmental health because they offer a rapid and sensitive mean of monitoring the impact of chemicals on living organisms. Enzymatic inhibition studies have been a very fruitful field for environmental monitoring applications. For example, the inhibition of acetylcholinesterase enzymatic activity by organophosphate and carbamate pesticides has been widely used in biomonitoring field for environmental neurotoxicant risk assessment [9,10,11,12,13,14,15].

The review focuses on the enzyme carbonic anhydrase (CA; EC 4.2.1.1) and discusses its potential as a novel biomarker in environmental monitoring and assessment. CA is a ubiquitous metallo-enzyme present in the bacterial, plant and animal kingdoms, which catalyses the reversible hydration of CO_2 _to produce H^+^ and HCO_3_^−^. Recently, a number of evidences have emerged regarding the effect of pollutants on carbonic anhydrase catalytic activity. These studies offer new perspectives in the application of this enzyme as biomarker in environmental biomonitoring [16].

## 2. Carbonic Anhydrase Enzyme: General Features

CA is a zinc metalloenzyme which plays key roles in a wide variety of physiological processes involving CO_2_ and HCO_3_^−^. Its activity is virtually ubiquitous in Nature. Five CA families, referred as α-, β-, γ-CA, δ, and ζ-CAs have been identified in animals, plants and bacteria [17]. The α-CAs are present in vertebrates, bacteria, algae and plants; the β-CAs are predominant in bacteria, algae and plants; the γ-CAs are mainly present in Archaea and some bacteria; the δ-CAs and ζ-CAs are only found in some marine diatoms [17].

The α-carbonic anhydrases are monomeric isoenzymes and are by far the best studied, being found in animals. In mammals at least 16 different CA isoforms were isolated and several novel isozymes have also been identified in non-mammalian vertebrates. In general, there are three distinct groups of CA isozymes within the α-CA gene family. One of these groups contains the cytoplasmic CAs, which includes mammalian CA I, II, III, V, VII and XIII. Another group of isozymes, termed the membrane-bound CAs, consists of mammalian CA IV, IX, XII, XIV and XV [18]. They are associated with the plasma membranes of many different tissue types [19]. The final group includes several very intriguing isozymes, CA VIII, X and XI, which are termed the CA-related proteins (CA-RP) [16]. These isozymes have lost classical CA activity—the hydration/dehydration of CO_2_—and have no known physiological function [20]. In animals the various CA isozymes are found in many different tissues and are involved in a number of different physiological processes, including bone resorption, calcification, ion transport, acid-base transport, and a number of different metabolic processes such as biosynthetic reactions (gluconeogenesis, lipogenesis, and ureagenesis).

The β-carbonic anhydrases are dimers, tetramers, or octamers and include the majority of the higher plant CA isoforms [21]. The γ-carbonic anhydrase is a homotrimer that has been found in the bacterium *Methanosarcina thermophila* [22]. The δ class has its prototype in the monomeric CA TWCA1 from the marine diatom *Thalassiosira weissflogii* [23,24]. The ζ-CAs are probably monomer with three slightly different active sites on the same protein backbone [25]. In algae, plants, and some bacteria CA isoforms play an important role in photosynthesis [26,27,28].

CAs catalyze the reversible hydration of carbon dioxide to bicarbonate and protons by means of a metal-hydroxide [Lig^3^M^2+^(OH)^−^] mechanism, although the α-CAs possess also other catalytic activities such as esterase, phosphatase, cyanate/cyanamide hydrase, *etc.* [29,30,31]. In the α-, γ-, and δ-CA classes, Lig^3^ is always constituted by three His residues. The metal (M) is ZnII for all classes. The zinc atom is in the +2 state and is located in a cleft near the centre of the enzyme. The role of zinc in carbonic anhydrase is to facilitate the deprotonization of water with the formation of the nucleophilic hydroxide ion, which in turn can attack the carbonyl group of carbon dioxide to convert it into bicarbonate.

Besides zinc, other metals have demonstrated to be physiologically relevant cofactor for some CAs. The γ-CAs can use FeII as their metal [32], while the ζ-CA naturally uses Cd^2+^ [33,34,35].

## 3. Carbonic Anhydrase and Heavy Metals

In recent years one of the most worrying class of chemical contaminants in term of toxicological risk for human and wildlife is represented by heavy metals. Pollution by trace metals is a world-wide problem because of the persistency and continuing accumulation of metals in the environment [36,37]. Heavy metals may enter the organism through food, water, air, or absorption through the skin and exert known toxic effects on living organisms [38,39,40]. As a result of mining, waste disposal and fuel combustion the environment is becoming increasingly contaminated by heavy metals.

Besides the contribution of same heavy metals as cofactors in the catalytic activity of carbonic anhydrase, several heavy metals were demonstrated to inhibit CA activity *in vitro* in a variety of animals, both vertebrates and invertebrates. Christensen and Tucker [41] demonstrated for the first time carbonic anhydrase to be inhibited by heavy metals in fish. Later works revealed the sensitivity of CA to trace metals both in lower vertebrates and mammals. Lionetto *et al*. [42,43] found a significant tissue-specific inhibition of CA by cadmium in the intestine and gills of the European eel, *Anguilla anguilla*. The cytosolic CA activity present in the gills was much more sensitive to the heavy metal than the CA cytosolic isoform in the intestine (IC_50_ in the gills: 9.979 × 10^−6^ M; IC_50_ in the intestine: 3.64 × 10^−5^ M). In particular in the intestine, where both a cytosolic and a membrane-bound isoforms were detected, the inhibitory effect of cadmium was more pronounced on the cytosolic than the membrane-bound CA. The cadmium inhibition showed also a time-dependence with a delay of at least 10 min and 30 min for the cytosolic isoform and the membrane-bound isoform respectively. As suggested by the authors, the inhibition delay could be due to the time required by cadmium for displacing the metal (zinc) associated with the enzyme, giving an inactive Cd-substituted CA. Cadmium is a bivalent metal, similar in many respects to zinc: both are in the same group of the periodic table, contain the same common oxidation state (+2), and when ionized have almost the same size. Due to these similarities, cadmium can replace zinc in many biological systems. Moreover, the delayed inhibition of the membrane-bound CA with respect to the cytosolic isoform was explained by a more difficult access of cadmium to the active site.

In aquatic invertebrates Skaggs *et al*. [44] demonstrated a significant *in vitro* inhibition of cytosolic CA by Ag^+^, Cd^2+^, Cu^2+^ and Zn^2+^ in the gills of the crabs *Callinectes sapidus *and *Carcinus maenas. *The inhibition was species-specific with a binding affinity for the metals one thousand times weaker in *C. maenas *with respect to *C*. *sapidus*.

Also in humans CA activity showed a marked sensitivity to heavy metal exposure. Ekinci *et al*. [45] demonstrated the inhibition of the cytosolic HCA-I and HCA-II by lead, cobalt and mercury. Lead was a non-competitive inhibitor for HCA-I and competitive for HCA-II, cobalt was competitive for HCA-I and non-competitive for HCA-II and mercury was uncompetitive for both HCA-I and HCA-II. Lead was the best inhibitor for both HCA-I and HCA-II.

The species-specificity observed in the inhibition of carbonic anhydrase activity by heavy metals in both humans and other animals can arise from the fact that heavy metals bind to CA not at the specific catalytic site of CO_2 _hydration but nearby in a pocket that is termed the “proton shuttle” [46]. Therefore, it is plausible that structural differences in CA protein isoforms could result in different metal-binding affinities in different species.

Considering the pivotal role played by carbonic anhydrase in organisms’ physiology, these results point out the interest for the study of carbonic anhydrase response to heavy metal exposure for both human and environmental health monitoring.

Lionetto *et al*. [47] investigated CA activity inhibition by heavy metals in the filter feeding mussel *Mytilus galloprovincialis*, widely used as sentinel organism in pollution monitoring programs [48]. Mantle CA activity was significantly inhibited following *in vitro* and *in vivo* exposure to cadmium. Considering the important role of CA in the calcification process, the inhibitory effect of cadmium on mantle CA activity can explain results previously obtained by Soto *et al*. [49], who observed a significant decreased in shell growth of *M. galloprovincialis* exposed to heavy metals. Interestingly, the sensitivity of CA to heavy metals in *M. galloprovincialis* appeared to be tissue-specific. While in mantle cadmium induced a significant inhibition of CA activity, in digestive gland the metal was demonstrated to increase CA activity and expression following *in vivo* exposure [50]. Moreover, digestive gland CA activity showed a weak sensitivity to cadmium exposure *in vitro, *since only high concentration of CdCl_2_ (10^−4^ and 10^−3^ M) were able to exert a significant inhibition. This is the first time that CA activity and protein expression are demonstrated to be enhanced by the exposure to the trace element cadmium in animals, opening new perspective in the comprehension of the functioning and regulation of this enzyme. Carbonic anhydrases from the microalgae *Chlamydomonas reinhardtii* [51] and *Thalassiosira weissflogii* [52,53] are the only other examples reported in nature of CA activity increased by cadmium exposure. Evidence of *in vivo* utilization of Cd in CA has been found in microalgae [52,53,54]. In these organisms Cd is able to substitute to Zn at the active site, without impairing the catalytic activity. In *Thalassiosira weissflogii* a cadmium-containing CA was found to be expressed during zinc limitation [33,34]. This cadmium CA (CDCA1) which naturally uses Cd as its catalytic metal [24,34] has been ascribed to a novel ζ-CA class (see above). Genes coding for similar proteins have been identified in other cultured diatoms [55]. In mussel (*Mytilus galloprovincialis*) digestive gland western blotting analysis clearly demonstrated the enhancement of CA protein expression following cadmium exposure, in parallel with an increase in the enzymatic activity (about 40% increase after two weeks of exposure) [50]. If the new synthesized enzyme is a Cd-CA is not possible to say at the moment and further research is needed to address this intriguing issue. The studies on mussels demonstrated that in the same organism different CA isoforms can show completely different responses to metal exposure. This aspect needs to be carefully considered during application of CA analysis in biomonitoring.

## 4. Carbonic Anhydrase and Organic Chemical Pollutants

As regards the sensitivity of carbonic anhydrase to organic chemical pollutants, most of the available data were obtained from pesticides, which represent one of the most worrying classes of chemical contaminants in term of toxicological risk for humans and wildlife. In the last two decades the interest in the toxicity of pesticides has been extended, including not only the direct effects on man but also the far more subtle effects that pesticides exert on natural biota.

CA purified from erythrocytes of the sturgeon *Acipenser gueldenstaedtii *was demonstrated to be inhibited *in vitro* by several widely used pesticides (2,4-dichlorophenol, dithiocarbamates, parathion and carbaryl) [56]. The dithiocarbamates were low micromolar CA inhibitors (IC_50_ of 16–18 µM), whereas the other pesticides inhibited the enzyme with IC_50_s in the range of 102–398 µM. In addition other commonly herbicides and fungicides, used in agriculture (imazethapyr, 2,4-D dimethylamine salt, glyphosate isopropylamine salt and propamocarb HCl) were assayed as inhibitors of human erythrocyte carbonic anhydrase (hCA-I, hCA-II isozymes) with imazethapyr being the most effective (IC_50_: 9.3 × 10^−5^ M) [57]. Işık *et al*. [58] investigated the effects of various pesticides such as nuarimol, fenarimol, parathion-methyl and 2,4-dichlorophenoxy acetic acid on CA activity from some freshwater and seawater fish erythrocytes, and found that the pesticides used inhibited the CA activity from different fish species to various degrees. It was found that I_50_ values for nuarimol, fenarimol, parathion-methyl and 2,4-dichlorophenoxy acetic acid pesticides were 0.38, 0.55, 2.9 and 2.72 mM for *C. carpio *CA, 0.28, 0.59, 2.45 and 1.73 mM for *Barbus barbus *CA, 0.23, 0.51, 1.77 and 1.26 mM for *O. mykiss *CA, 0.20, 0.18, 0.62 and 0.65 mM for *Scorpaena porcus *CA, 0.38, 0.37, 3.19 and 2.67 mM for *Diplodus vulgaris *CA, respectively. The most effective inhibitor of CA enzyme within pesticides used was *deltamehtrin*. This conclusion was also confirmed by the study of Doğan *et al*. [59] on CA activity obtained from the erythrocytes of *Oncorhynchus mykiss* and *Cyprinus carpio* fishes. *Deltamehtrin *demonstrated to be an effective CA inhibitor also in *in vivo* exposure, as demonstrated on CA purified from rainbow trout gills [60].

As observed for CA inhibition by heavy metals, a high species-specificity was also found in the inhibition of CA by pesticides. It is possible that the different sensitivity observed could reflect differences in binding affinity of the pesticides to the enzyme, as a result of species-specific isoforms.

The sensitivity of CA to organic chemical pollutants other than pesticides was demonstrated by Lionetto *et al*. [16], who found CA II extracted from bovine erythrocytes to be highly sensitive, not only to the carbamate pesticide carbaryl, but also to the polychlorinated biphenyl (PCB) arochlor, showing an inhibition of 34.4% at the concentration of 10 ng·L^−1^. Considering the high homology between the bovine and human carbonic anhydrase II , these results can be extrapolated to humans. Moreover, they suggest the interest for the study of carbonic anhydrase response to organic chemical pollutant exposure for both human and environmental health monitoring.

## 5. Potential Application of Carbonic Anhydrase as Biomarker in Environmental Monitoring

The studies carried out to date have revealed the sensitivity of CA from a wide variety of tissues in numerous species to different types of pollutants. They open new perspectives for the potential application of CA as a pollution biomarker in environmental biomonitoring and assessment. However, some important issues need to be addressed in future studies. A crucial question is that any biological response to chemical pollutant exposure must meet a number of characteristics to be applied successfully as a biomarker in environmental biomonitoring. These characteristics include whether the response is easy to measure, whether it responds in a dose-dependent manner to pollutants, and whether it is sensitive. In addition the variability in the biomarker response due to natural variation should be understood (*i.e.*, season, temperature, sex, weight, and handling). Finally, a key issue is that the biomarker response should exhibit a link to adverse effects at the organism level including processes such as growth, reproduction and mortality.

CA activity can be measured by a number of methods. The most widely used is the electrometric method early described by Wilbur and Anderson [61] and further modified by other authors (see for example [19]). The electrometric method is based on the measurement of the rate of pH decrease in the reaction medium containing the enzyme and its substrate (CO_2_). This method is easy to use, employs relatively inexpensive equipment and the results are accurate and quantitative [62]. These features make CA activity measurement suitable for in in field biomarker application in sentinel organisms. 

The *in vitro* and *in vivo* studies carried out to date reveal a dose-response sensitivity of CA activity to a number of pollutants, both heavy metals and xenobiotics, suggesting the possible use of CA activity alterations as general pollutant biomarker. However, the high specie-specificity and tissue-specificity of the observed responses suggest the need of a thorough knowledge of the pollutant induced CA alterations in the specific bioindicator species utilized.

CA plays a key role in a number of physiological processes both in humans and wildlife. Therefore, it is reasonable to think that any impairment of carbonic anhydrase activity by environmental pollutants could put the survival of the organisms at risk, suggesting the relevance of this ubiquitous enzyme as environmental biomarker. However, very few information are available to date on the direct relationships between chemical exposure, CA response and health adverse effects. Although it is known that CA activity is virtually always expressed in excess of the physiological process that it supports and so it is never the rate-limiting step in any physiological or biochemical process, some experimental evidences highlight the impairment of some physiological functions by pollutant-induced CA inhibition. For example, in the estuarine crab *Chasmagnathus granulata* Vitale *et al*. [63] related the inhibition of gill CA by cadmium to a decrease of blood Na^+^ concentration and, in turn, to the impairment of ionic regulation of the animal particularly at lower salinities. In *Mytilus galloprovincialis* Soto *et al*. [49] observed a significant decrease in shell growth in *M. galloprovincialis* exposed to heavy metals. This result can be explained by the inhibition of mantle CA, found by Lionetto *et al*. [47] during *in vivo* exposure experiments. The early work of Peakall [64] demonstrated a significant decrease in eggshell weights associated with significantly reduced oviduct carbonic anhydrase activity (which is involved in the secretion of the calcareous eggshell) in ringed turtle doves injected intraperitoneally with 150 mg/Kg p,p'*-*DDE. However, further research is needed to betterunderstand the *in vivo* inhibition of CA by pollutants and its relationship with the impairment of some physiological functions and in general with the health status of the organisms. 

Biological responses are influenced by a number of natural environmental factors. A number of studies on the biomarker approach pointed out the need to incorporate the effects of abiotic (temperature, salinity, pH, *etc.*) and biotic factors (age, gender, reproduction cycle, *etc*.) to a correct interpretation of biomarker responses [65,66]. To date the natural variability of CA activity in different species is almost completely unknown and requires future investigation for a correct use of CA as biomarker in biomonitoring. It is well understood that the biological responses of an organism to pollutant exposure can be various because of the variety of pollutants that may be present simultaneously in the environment. Thus, a suite of biomarkers is required to be effectively applicable in biomonitoring programmes to better understand the complex stress syndrome induced in the organisms by environmental pollutant exposure. Recently, CA measurements in field are beginning to be successfully included in multi-biomarker approaches on bioindicator organisms in environmental biomonitoring and assessment. The measurement of this enzyme can contribute to better appreciate the pollutant induced stress syndrome in living organisms because of the involvement of CA in a number of physiological processes (including pH homeostasis, osmoregulation, and calcification). For example the water quality in three reservoirs along the Paraiba do Sul River (located at a very densely inhabited region of Brazil in a high industrialized area) was monitored through physiological, morphological, genetic and biochemical biomarkers, including carbonic anhydrase activity inhibition, in the bioindicator fish *Pimelodus maculates* [67].

Recent studies carried out on corals have suggested inhibition of CA activity in these organisms as potential biomarker of exposure to environmental chemical stress. CA activity has been demonstrated to be inhibited by heavy metal exposure with consequent impairment of the calcification process and inhibition of coral growth [68]. In an era of climate change and ocean acidification the study of carbonic anhydrase in coral contribute to understand biological effects of pollution exposure to these keystone tropical organisms [68].

Moreover, the applicability of carbonic anhydrase expression in a multimarker approach in the bioindicator organism *M. galloprovincialis *was recently demonstrated by Caricato *et al*. [50,69] in a study aimed to evaluate the potential ecotoxicological risk in a coastal marine area exposed to industrial and urban impact. Mussels exposed for 30 days to an anthropogenic impacted site showed a significant increase in digestive gland CA activity and expression with respect to animals exposed for 30 days in the control site [50]. This response paralleled metallothionein induction (specific biomarker of heavy metal exposure) in the same animals. The results clearly demonstrate for the first time that CA protein expression in mussel digestive gland increases during field heavy metal exposure condition, and document its applicability in a multibiomarker approach. Mussel digestive gland is characterized by a very developed endo-lysosomal system [70]. CA, catalyzing the H^+ ^production from metabolic CO_2_, can provide the H^+^ necessary for the lysosome acidification. Considering this possible functional relation between CA and the lysosomal compartment, the observed CA upregulation induced by metal exposure in mussel digestive gland could be related to the well-known activation of the lysosomal system widely described in the digestive gland of pollutant exposed mussels [70]. In this regard the utility of measuring digestive gland CA expression in a multibiomarker approach on mussel is to help strengthen and complement the information provided by the study of the lysosomal system.

## 6. Conclusions

In recent years the disclosed sensitivity of CA to chemical pollutants has opened a new future for the potential application of this ancient enzyme in biological monitoring and assessment both in humans and wildlife. The ubiquitous nature of CA in living organisms makes this enzyme a particularly versatile biomarker that can be used to investigate pollutant effects in many trophic levels and in many different environments. It already demonstrates some of the necessary characteristics for successful application as an effective biomarker in monitoring and assessment programs. This includes an evaluation of pollutant-induced stress at the biochemical-cellular level in an easy, sensitive, and inexpensive way in addition to applicability in the laboratory and the field. However, further studies are required in order to better characterize the responses of CA to pollutant exposure in living organisms and implement the potential of this enzyme in environmental monitoring and assessment.
